# Prognostic significance of CD155 mRNA expression in soft tissue sarcomas

**DOI:** 10.3892/ol.2013.1280

**Published:** 2013-04-02

**Authors:** SATORU ATSUMI, AKIHIKO MATSUMINE, HIDEMI TOYODA, RUI NIIMI, TAKAHIRO IINO, AKIHIRO SUDO

**Affiliations:** 1 Departments of Orthopedic Surgery, Mie University Graduate School of Medicine, Tsu, Mie 514-8507, Japan; 2 Pediatrics, Mie University Graduate School of Medicine, Tsu, Mie 514-8507, Japan

**Keywords:** CD155/PVR/Necl-5, soft tissue sarcoma, local recurrence, biomarker, prognosis

## Abstract

CD155 was initially identified as a receptor for poliovirus. Several studies have demonstrated that CD155 overexpression in cancer cells is significant in their migration, invasion, proliferation and metastasis. The objective of the present study was to investigate the correlation between *CD155* expression and the clinical aggressiveness of soft tissue tumors. The *CD155* expression levels in 43 surgically-resected soft tissue tumors were evaluated using the quantitative real-time polymerase chain reaction (PCR). The clinicopathogical factors affecting the expression levels of *CD155* mRNA were investigated and the association between the expression levels of *CD155* and patient prognosis was identified. The *CD155* expression level was not correlated with the patient gender, site of the primary tumor, tumor depth, tumor size or presence of distant metastasis at presentation, but was correlated with patient age (Fisher’s exact test). The local recurrence-free survival rate for patients with a high *CD155* expression level was observed to be significantly poorer compared with that of patients with low *CD155* expression levels (P=0.0401). Moreover, a multivariate analysis indicated that a high *CD155* expression level was an independent adverse prognostic factor for local recurrence-free survival (hazard ratio, 6.369; P=0.0328). The present study therefore suggests that the expression level of *CD155* is a useful marker for predicting the local recurrence of soft tissue tumors.

## Introduction

Soft tissue sarcoma is a malignant mesenchymal neoplasm with an incidence of ∼1% among all human malignancies (1). At presentation, numerous patients are already in the advanced stages of the disease, thus limiting the role of surgery as a curative treatment modality. Despite major advances in the treatment of soft tissue sarcomas, approximately one-quarter of all patients show a poor response to conventional therapy, including surgery, chemotherapy and radiotherapy (2–4). Several clinical studies have provided useful information with regard to the biological behavior of soft tissue sarcoma, including the effect of such factors as histological grading, tumor size and tumor location (2,3,5). However, improved molecular biomarkers for the accurate prediction of the biological potential for progression are required, as treatment decisions should be based on tissue-based morphological and biological indicators (6). We previously revealed that reduced decorin expression (7), over-expression of hypoxia-inducible factor (HIF)-1α (8) and high serum C-reactive protein levels (9) were useful biomarkers of the aggressiveness of soft tissue sarcoma.

CD155/poliovirus receptor (PVR) was initially identified as a receptor for poliovirus, as a result of the observation that anti-CD155 antibodies were able to block viral entry into cells (10,11). CD155 is also known as nectin-like molecule-5 (Necl-5) and is one of the Necl family members (12). CD155/PVR/Necl-5 is preferentially localized at the leading edge of moving cells and promotes cell movement and proliferation (13). The physiological roles of CD155/PVR/Necl-5 have also been reported. At the leading edge of the moving cells, CD155/PVR/Necl-5 enhances cell movement and proliferation cooperatively with activated integrin α_v_β_3_ and growth factor receptors, such as the platelet-derived growth factor (PDGF) receptor (14). When moving cells come into contact with each other, the initial cell-cell contact that occurs via the trans-interaction of CD155/PVR/Necl-5 with nectin-3 and integrin α_v_β_3_ remains active. The trans-interaction of CD155/PVR/Necl-5 and nectin-3 is transient. Nectins and cadherins interact with each other to form adherence junctions once CD155/PVR/Necl-5 is endocytosed in a cadherin-dependent manner, which leads to its disappearance from the cell surface (15). Thus, the expression of CD155/PVR/Necl-5 is downregulated when cultured cells become confluent, resulting in the suppression of cell movement and proliferation (16).

CD155 is expressed at low levels in a number of cell types of epithelial origin and is overexpressed in various carcinomas with epithelial and neurological origins, including colorectal carcinoma (17), breast carcinoma, neuroblastoma and glioblastoma (18,19). The downregulation of CD155 in cancer cell lines decreases their migration (20,21), proliferation (22) and metastasis (23). It has also been shown that CD155 is involved in the development of colitis-associated cancer by upregulating colonic mucosal cell proliferation (24). Nakai *et al* showed that the overexpression of CD155 has clinical significance for the prognostic evaluation of patients with primary pulmonary adenocarcinoma (25).

We have previously shown that CD155 was widely expressed in various human bone and soft tissue sarcoma cell lines (26), but there have so far been no studies describing the clinicopathological implications of CD155 in soft tissue sarcomas. The present study investigated whether the *CD155* gene expression level in surgically resected human primary soft tissue sarcoma tissues has prognostic significance.

## Patients and methods

### Patient details

The details of the clinicopathological features of the patients are shown in Table I. The present study involved 43 patients (24 males and 19 females). The median age of the patients was 45.6 years (range, 0–85 years) and the median follow-up was 76.5 months (range, 8–154 months). All patients underwent complete tumor resection with a wide margin during the initial surgery at the Department of Orthopedic Surgery, Mie University Graduate School of Medicine (Tsu, Japan). All samples were of primary lesions. At the final follow-up, 20 patients had been continuously disease-free, 2 showed no evidence of disease, 4 were living with the disease, 16 had succumbed to the disease and 1 had succumbed to an unrelated cause. The study was approved by the Ethics Committee of Mie University Graduate School of Medicine.

The pathological diagnoses were made by well-trained pathologists. Immunohistochemical staining was used in all cases for the diagnosis of malignant fibrous histiocytoma (MFH; n=16), synovial sarcoma (n=9), malignant peripheral nerve sheath tumors (MPNST; n=4), rhabdomyosarcoma (n=3), clear cell sarcoma (n=2), alveolar soft part sarcoma (ASPS; n=2), Ewing’s sarcoma (n=1), extraskeletal myxoid chondrosarcoma (n=1), leiomyosarcoma (n=1), malignant granular cell tumor (n=1), infantile fibrosarcoma (n=1), mixofibrosarcoma (n=1) or solitary fibrous tumor (n=1), according to the World Health Organization classification. Patients who presented with recurrent sarcomas following inadequate treatments at the initial hospital were excluded from the present study. Patients who had distant metastases at the time of the initial treatment were included for the analyses evaluating the overall survival and local recurrence-free survival. However, they were excluded from the analysis of the metastasis-free survival.

### Preparation of tissue samples

The tissue specimens were obtained from patients who underwent surgical resection or an open biopsy at the Department of Orthopedic Surgery, Mie University Graduate School of Medicine, subsequent to obtaining informed consent according to the institutional review board guidelines. Tissue samples were immediately collected from either biopsied or excised tumor tissue and then either snap-frozen in liquid nitrogen (for RNA extraction) or fixed for 24 h in 10% buffered formalin solution and embedded in paraffin (for the histological analysis). The tumor grade was assessed according to the updated version of the FNCLCC system based on the tumor differentiation, mitotic count and necrosis (4).

### Total RNA extraction and quantitative real-time polymerase chain reaction (PCR)

Total RNA was extracted from each sample using ISOGEN (Nippon Gene, Tokyo, Japan), according to the manufacturer’s instructions. The RNA was then reverse-transcribed into cDNA using the First Strand cDNA Synthesis kit (Roche Applied Science, Mannheim, Germany). The TaqMan^®^ Gene Expression Master Mix and the TaqMan Gene Expression Assay (Applied Biosystems, Foster City, CA, USA) were used to quantitatively analyze the expression of the genes, including glyceraldehyde-3-phosphate dehydrogenase (GAPDH) and CD155. Real-time quantitative PCR amplifications were performed using an ABI PRISM^®^ 7000 Sequence Detection System (Applied Biosystems). GAPDH was used as an endogenous housekeeping gene for normalization. Standard curves were generated using cDNA samples from HeLa cells. The relative expression levels of each target gene were indicated by calculating the ratio to the expression levels in the HeLa cells. All assays were performed in triplicate and repeated three times. The samples were regarded as having high CD155 expression if the CD155 mRNA expression levels were higher than those of the HeLa cells, since HeLa cells have been used as standard samples in a number of studies to measure the CD155 expression level (10,18,19).

### Statistical analysis

Fisher’s exact test was used to analyze the associations between the clinicopathological variables. The tumor grade was excluded from the statistical analysis, since there were only two low grade cases included in the present study. The overall survival was defined as the time from the initial treatment to the date of mortality attributed to the neoplasm. The local recurrence-free survival was defined as the time from the initial treatment to the date of clinically documented local recurrence. The metastasis-free survival was defined as the time from the initial treatment to the date of clinically documented distant metastasis. To investigate the prognostic value of the expression levels, Kaplan-Meier survival analyses and log-rank tests were performed. The variable effects on the local recurrence-free survival time were investigated using the Cox proportional hazards regression model. P<0.05 was considered to indicate a statistically significant difference. The data analysis was performed using the StatView statistical software package, version 5.0 (SAS Institute, Cary, NC, USA).

## Results

### CD155 expression in various soft tissue sarcomas

**Figure 1:**
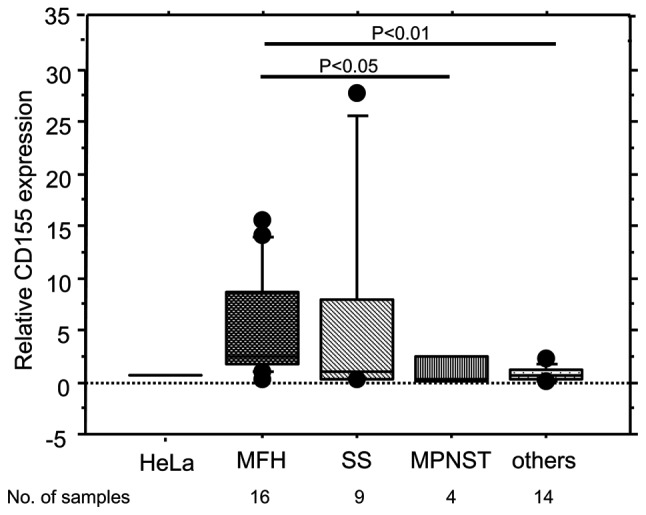
*CD155* expression in various soft tissue sarcomas. The relative *CD155* expression was assessed by multiplex real-time quantitative PCR. The expression level of *CD155* was significantly higher in MFH than in malignant peripheral nerve sheath tumors (MPNST; P<0.05). The expression level of *CD155* was also significantly higher in MFH compared with the other tumors (P<0.01), including rhabdomyosarcoma (n=3), clear cell sarcoma (n=2), alveolar soft part sarcoma (ASPS) (n=2), Ewing’s sarcoma (n=1), extraskeletal myxoid chondrosarcoma (n=1), leiomyosarcoma (n=1), malignant granular cell tumor (n=1), infantile fibromatosis (n=1), mixofibrosarcoma (n=1) and solitary fibrous tumor (n=1). PCR, polymerase chain reaction; MFH, malignant fibrous histiocytoma; MPNST, malignant peripheral nerve sheath tumors; SS, synovial sarcoma.

To evaluate the expression of *CD155* mRNA in various soft tissue sarcomas, real-time quantitative PCR analysis was performed (Fig. 1). The expression level of *CD155* was significantly higher in MFH than in MPNST (P<0.05). The expression level of *CD155* was also significantly higher in MFH compared with the other tumor types (P<0.01), including rhabdomyosarcoma (n=3), clear cell sarcoma (n=2), alveolar soft part sarcoma (ASPS) (n=2), Ewing’s sarcoma (n=1), extraskeletal myxoid chondrosarcoma (n=1), leiomyosarcoma (n=1), malignant granular cell tumor (n=1), infantile fibromatosis (n=1), mixofibrosarcoma (n=1) and solitary fibrous tumor (n=1). Furthermore, the *CD155* expression was increased in sarcomas with spindle-shape cells, such as MFH and synovial sarcoma, compared with those with different morphologies.

### Correlation between the relative expression levels of CD155 and various clinicopathogical factors

Since HeLa cells have been used in a number of investigations to measure the *CD155* expression level as a standard sample (10,18,19), the *CD155* expression level of HeLa cells was used as a cut-off value to divide the patients into groups with high and low *CD155* expression levels. According to this cut-off value, 24 patients exhibited high *CD155* expression levels and 19 exhibited low expression levels. The correlations between the *CD155* expression level and various clinicopathological factors are shown in Table I. A significant correlation was observed between the *CD155* expression level and age, but no significant correlation was observed between the *CD155* expression level and the other clinicopathological factors, including gender, site of the primary tumor, tumor depth, tumor size and distant metastasis at presentation (M1) (Fisher’s exact test).

### Prognostic analysis

**Figure 2:**
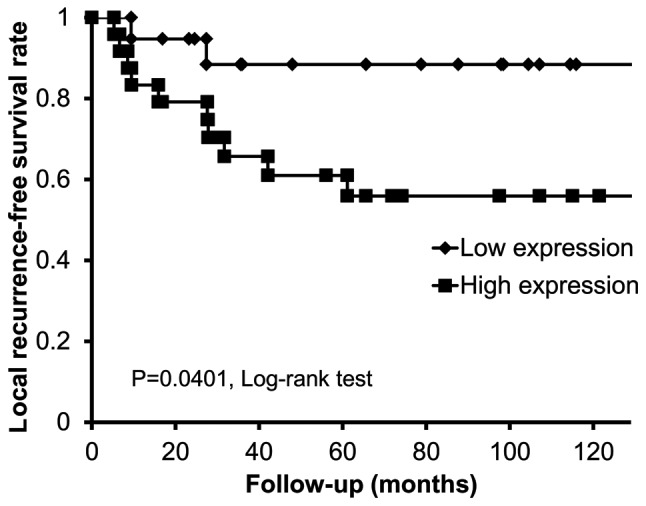
Cumulative local recurrence-free survival of soft tissue sarcoma patients with high CD155 expression levels compared with patients with low expression levels. Kaplan-Meier curves demonstrated that the local recurrence-free survival rate in patients with low expression levels of *CD155* mRNA was significantly reduced.

**Figure 3:**
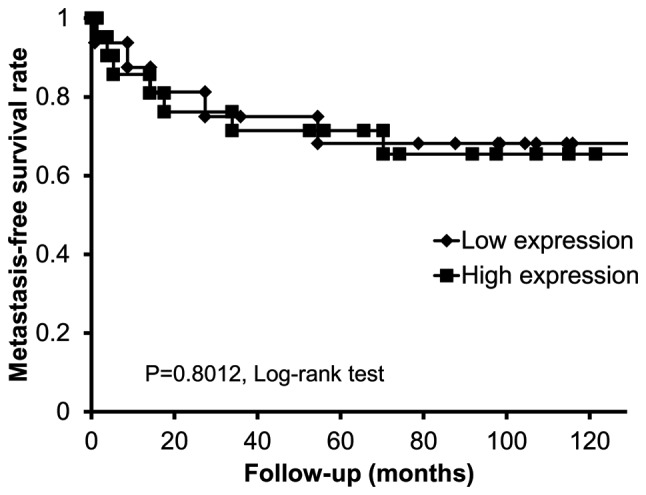
Cumulative metastasis-free survival of patients with high CD155 expression levels compared with patients with low expression levels in soft tissue sarcoma. Kaplan-Meier curves demonstrated that there were no statistically significant differences in the metastasis-free survival rates between the patients with higher expression levels of *CD155* and those with lower expression levels.

**Figure 4:**
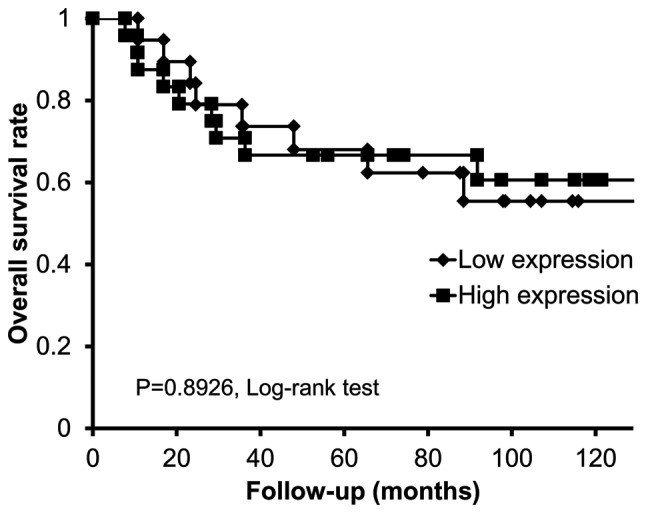
Cumulative overall survival of soft tissue sarcoma patients with high *CD155* expression levels compared with patients with low expression levels. Kaplan-Meier curves demonstrated that there were no significant differences in the overall survival rates between the patients with higher expression levels of *CD155* and those with lower expression levels.

Next, the local recurrence-free survival, metastasis-free survival and overall survival of the patients showing high *CD155* expression levels were compared with those of patients showing low *CD155* expression levels. Kaplan-Meier survival analyses and log-rank tests were performed for all patients (Figs. 2, 3 and 4). A univariate analysis demonstrated that the level of *CD155* expression was significantly associated with local recurrence-free survival (P=0.0401; Table II and Fig. 2). However, the level of *CD155* expression was not significantly associated with the metastasis-free survival (P=0.801; Table III and Fig. 3) or overall survival (P=0.892; Table IV and Fig. 4). The soft tissue sarcoma patients with a higher *CD155* expression exhibited poorer local recurrence-free survival compared with patients with lower *CD155* expression levels. The patients with distant metastasis at presentation (M1) showed a poorer overall survival compared with non-metastatic patients (M0). However, no significant differences were observed between local recurrence-free survival, metastasis-free survival or overall survival and any of the other prognostic factors, including patient age, gender, tumor size and tumor depth (Tables II, III and IV).

Multivariate analysis demonstrated that the expression level of *CD155* was the only independent prognostic factor for local recurrence-free survival (hazard ratio, 6.369; 95% CI, 1.163–34.482; P=0.0328; Table V). The expression of *CD155* was not an independent prognostic factor for the metastasis-free survival or overall survival (data not shown). There were no significant differences in local recurrence-free survival with regard to the other prognostic factors, including patient age, gender, tumor size, tumor depth and distant metastasis at presentation (Cox’s multivariate analysis; Table V).

## Discussion

Soft tissue sarcoma is a malignant mesenchymal neoplasm with an incidence of ∼1% among all human malignancies (1). Despite major advances in the treatment of soft tissue sarcomas, approximately one-quarter of all patients show a poor response to conventional therapy (2–4). Biomarkers that are able to predict which patients are at high risk are extremely important, as such biomarkers are useful for determining whether adjuvant therapies, including radiation and chemotherapy, should be used. In patients with soft tissue sarcomas, the tumor size, tumor location and histological grade have been shown to be important prognostic factors (10). However, these prognostic factors do not always reliably predict the outcome of the patient. In the present study, it was demonstrated that high levels of CD155 were associated with local recurrence in patients with soft tissue sarcomas.

We initially considered that CD155 may be expressed in neurogenic sarcomas, such as MPNST, since the upregulation of CD155 expression in previous studies was mainly observed in neuroectodermal malignancies (e.g. glioblastoma, medulloblastoma or neuroblastoma) (27,28,29). However, CD155 expression was observed in various histological types of sarcoma. The expression of *CD155* was particularly increased in the spindle cell sarcomas, including MFH and synovial sarcoma. Upregulated expression levels of CD155 have been reported in various types of cancer, including colorectal carcinoma (17) and lung adenocarcinoma (25). The present study is the first to show that *CD155* overexpression in soft tissue sarcoma tissue samples is associated with the clinical outcome of the patients.

In the present study, patients with high expression levels of *CD155* had a significantly shorter local recurrence-free survival compared with those with low expression levels in the univariate and multivariate analyses. A previous study of lung cancer patients showed that CD155 overexpression was correlated with lymph node metastasis and the TNM stage and that the disease-free survival of patients with CD155 overexpression was significantly lower than in patients without CD155 overexpression (25).

There have been numerous *in vitro* experimental studies that have elucidated potential mechanisms underlying the adverse effects of CD155 overexpression on patient prognosis. For example, CD155/PVR/Necl-5 localizes at the leading edge of migrating cells, together with integrin α_v_β_3_ and the PDGF receptor (14,23,30), and acts as mediator of the motility and adhesion of migrating cells. Blocking CD155/PVR/Necl-5 with anti-CD155/PVR/Necl-5 antibodies (20) and the knockdown of CD155/PVR/Necl-5 by RNAi (27) resulted in decreased migration of glioblastoma cells. The knockdown of CD155/PVR/Necl-5 in glioblastoma cells also decreased the matrix metalloproteinase-2 (MMP-2) expression and activity and resulted in markedly decreased cell invasion (31). NIH3T3 cells transformed by oncogenic Ki-Ras (V12Ras-NIH3T3 cells) exhibited overexpression of CD155/PVR/Necl-5 compared with wild-type NIH3T3 cells and had enhanced motility as a result of the trans-interaction with nectin-3 (21). Thus, CD155/PVR/Necl-5 has a key role in tumor cell invasion and migration. Moreover, CD155/PVR/Necl-5 is involved in regulating cell proliferation, since CD155/PVR/Necl-5 enhanced the PDGF-induced activation of the Ras-Raf-MEK-ERK signaling cascade and shortened the G_0_/G_1_ phase of the cell cycle in NIH3T3 cells (22). Thus, we suggest that CD155 overexpression is also involved in the local invasion and migration of aggressive soft tissue sarcoma. Together, these findings indicate that CD155 may be a useful biomarker for soft tissue sarcoma.

The chief limitation of the present study is the small number of samples examined. As is always the case in studies concerning soft tissue sarcoma, a large number of samples could not be collected due to the rarity of the tumors. In addition, soft tissue sarcoma has a variety of pathological classifications. As a result, a statistical analysis could not be performed for each pathological classification due to the small number of samples. To improve the efficacy of using the *CD155* expression level as a biomarker, it is necessary to investigate a larger number of samples. Moreover, prospective longitudinal sample collection from numerous patients (for example, pre-operatively, immediately after surgery and at the time when the sarcoma recurs), are likely to be indispensable for improving the reliability of using CD155 expression as a biomarker.

Poliovirus has been shown to selectively target cells with human CD155 (32,33). Several studies have also shown that live-attenuated poliovirus induces apoptotic cell death in tumors, including gliomas (28) and neuroblastomas (29), through the interaction with CD155 *in vitro* and *in vivo*. We previously showed that live-attenuated poliovirus has the potential to treat soft tissue sarcomas expressing CD155 as a form of oncolytic virotherapy (26). We therefore suggest that soft tissue sarcomas with upregulated *CD155* expression may be candidates for oncolytic virotherapy using live-attenuated poliovirus.

In conclusion, the overexpression of *CD155* was observed in various types of soft tissue sarcoma, and upregulated *CD155* expression was a significant predictor of local recurrence. These results indicate that CD155 may be a candidate molecular marker that may be used to predict local recurrence and that also represents a promising target for oncolytic virotherapy using live-attenuated poliovirus for soft tissue sarcoma.

## Figures and Tables

**Table I:** Results of univariate analysis of the associations between the CD155 expression levels and clinicopathological variables.

	CD155 expression		

Variable	Low (n)	High (n)	P-value
Age (years)			
<50	15	9	
≥50	4	15	0.0125
Gender			
Male	12	12	
Female	7	12	0.538
Location			
Extremities	15	17	
Trunk	4	7	0.7279
Tumor size (cm)			
<5	3	5	
≥5	16	19	>0.9999
Depth			
Superficial	2	4	
Deep	17	20	0.8853
M factor			
M0	16	21	
M1	3	3	>0.9999

M0, non-metastatic patients; M1, patients with distastant metastases at presentation.

**Table II:** Results of the univariate analysis of the local recurrence-free survival of 43 patients with soft tissue sarcoma.

Variable	n	10-year local recurrence-free survival (%)^a^	P-value
CD155			
Low expression	19	88.4	
High expression	24	55.9	0.0401
Age (years)			
<50	24	70.7	
≥50	19	67.7	0.6129
Gender			
Male	24	74.0	
Female	19	63.3	0.624
Location			
Extremities	32	74.8	
Trunk	11	54.5	0.0666
Tumor size (cm)			
<5	8	85.7	
≥5	35	65.4	0.2764
Depth			
Superficial	6	66.7	
Deep	37	70.1	0.8422
M factor			
M0	37	69.2	
M1	6	83.3	0.909

a Calculated using Kaplan-Meier method. M0, non-metastatic patients; M1, patients with distastant metastases at presentation.

**Table III:** Results of the univariate analysis of the metastasis-free survival of 37 patients with soft tissue sarcoma.

Variable	n	10-year metastasis-free survival (%)^a^	P-value
CD155			
Low expression	16	68.2	
High expression	21	65.5	0.8012
Age (years)			
<50	19	68	
≥50	18	66.2	0.9897
Gender			
Male	21	71.1	
Female	16	61.1	0.3924
Location			
Extremities	28	70.5	0.3602
Trunk	9	55.6	
Tumor size (cm)			
<5	7	83.3	
≥5	30	63.2	0.2431
Depth			
Superficial	6	67.4	
Deep	31	62.5	0.894

a Calculated using Kaplan-Meier method.

**Table IV:** Results of the univariate analysis of the overall survival of 43 patients with soft tissue sarcoma.

Variable	n	10-year overall survival (%)^a^	P-value
CD155			
Low expression	19	55.4	
High expression	24	60.6	0.8926
Age (years)			
<50	24	60.9	
≥50	19	54.4	0.7039
Gender			
Male	24	60.8	
Female	19	54.1	0.7076
Location			
Extremities	32	64.3	
Trunk	11	36.4	0.1175
Tumor size (cm)			
<5	8	87.5	
≥5	35	52.1	0.1225
Depth			
Superficial	6	54.8	
Deep	37	83.3	0.319
M factor			
M0	37	67.8	
M1	6	0.0	<0.0001

a Calculated using Kaplan-Meier method. M0, non-metastatic patients; M1, patients with distastant metastases at presentation.

**Table V:** Results of the multivariate analysis of the local recurrence-free survival of 43 patients with soft tissue sarcoma.

Variable	Hazard ratio	95% confidence interval	P-value
CD155 (high vs. low expression)	6.369	1.163–34.482	0.0328
Age (≥50 vs. <50 years)	0.459	0.112–1.890	0.281
Gender (male vs. female)	0.743	0.222–2.488	0.629
Location (trunk vs. extremities)	3.906	0.969–15.873	0.148
Tumor size (≥5 cm vs. <5 cm)	4.808	0.573–40.000	0.0554
Depth (deep vs. superficial)	0.706	0.140–3.566	0.674
M factor (M1 vs. M0)	0.357	0.0295–4.310	0.417

M0, non-metastatic patients; M1, patients with distastant metastases at presentation.
